# Discontinuation of treatment and retreatment of neovascular age-related macular degeneration in the real-world: Bundang AMD cohort study report 5

**DOI:** 10.3389/fmed.2023.1204026

**Published:** 2023-07-10

**Authors:** Soo Chang Cho, Kyu Hyung Park, Sang Jun Park, Kwangsic Joo, Se Joon Woo

**Affiliations:** ^1^Department of Ophthalmology, Ewha Womans University College of Medicine, Ewha Womans University Mokdong Hospital, Seoul, Republic of Korea; ^2^Department of Ophthalmology, Seoul National University College of Medicine, Seoul National University Hospital, Seoul, Republic of Korea; ^3^Department of Ophthalmology, Seoul National University College of Medicine, Seoul National University Bundang Hospital, Seongnam, Republic of Korea

**Keywords:** anti-vascular endothelial growth factor injection, discontinuation of treatment, neovascular age-related macular degeneration, polypoidal choroidal vasculopathy, retreatment, typical neovascular age-related macular degeneration

## Abstract

**Introduction:**

This single-center retrospective cohort study investigated the incidence rate and risk factors for the discontinuation of anti-vascular endothelial growth factor (VEGF) injections and retreatment in typical neovascular age-related macular degeneration (tnAMD) and polypoidal choroidal vasculopathy (PCV) in the real-world setting.

**Methods:**

A total of 488 eyes with either tnAMD (*n* = 334) or PCV (*n* = 154) followed up for ≥3 years were analyzed. The discontinuation of treatment was defined as the cessation of anti-VEGF injections for 1 year or longer. Eyes with discontinuing treatment were subdivided into group A: eyes with stable responses (complete or incomplete resolution) and group B: those with no expectation of visual gain or poor response. The proportion and median time of discontinuation of treatment or retreatment were analyzed. The visual prognosis and the associated risk factors for the discontinuation of treatment or retreatment were also investigated.

**Results:**

The mean follow-up period was 8.1 ± 3.4 years. Of 488 eyes, discontinuation of the treatment occurred in 322 eyes (66.0%), and the median time to discontinuation was 1.5 years after the initial injection. Of 297 eyes with discontinuation of treatment excluding 25 eyes with vitrectomy or photodynamic therapy after the discontinuation of the injection, 277 eyes belonged to group A and the remaining 20 eyes belonged to group B. Of the 277 eyes discontinuing treatment with a stable response, 185 eyes (66.8%) were given retreatment. The median time to retreatment was 3.3 years after the discontinuation of the injections. PCV and the lower annual number of injections were the significant factors associated with discontinuation. Younger age, male gender, and PCV were the significant factors for the retreatment.

**Conclusion:**

Our long-term real-world study showed that two-thirds of eyes with neovascular age-related macular degeneration (nAMD) had the discontinuation of the anti-VEGF injections and two-thirds of eyes discontinuing treatment with stable responses experienced retreatment. Long-term follow-up and regular monitoring are needed to detect the recurrence.

## Introduction

Age-related macular degeneration (AMD) is a chronic progressive eye disease affecting the central retina and can cause vision loss (1). Approximately 90% of severe vision loss is related to neovascular AMD (nAMD) (2). There is no definite cure for nAMD. Based on the results from several clinical trials (3–7) and a Cochrane systematic review of the literature (8), intravitreal anti-vascular endothelial growth factor (VEGF) therapy is needed as a first-line treatment and is effective for nAMD. The visual prognosis in nAMD has been improved with the application of intravitreal anti-VEGF injections (9). In spite of the clinical relevance as the first-line therapy for nAMD, the enormously increased frequency of anti-VEGF injections has been a considerable burden to both healthcare providers and patients (10). Furthermore, prolonged use of anti-VEGF injections exposes patients to adverse effects including endophthalmitis and macula atrophy (11). In order to reduce the burden of anti-VEGF injections, guidelines for the discontinuation of intravitreal injections in nAMD with mild activity or cases of futility have been proposed (12, 13). On the other hand, there is an opinion that injections be continued to maintain the effects of the anti-VEGF (14). It remains unclear whether the discontinuation of anti-VEGF injections is effective to reduce the burden while maintaining the efficacy of the treatment (15).

There have been some studies on treatment suspension in nAMD. One study reported a 24-month natural course of patients with nAMD discontinuing treatment despite persistent or recurrent fluid (16). The study reported an apparent visual deterioration in patients discontinuing treatment. The study also found that the presence of intra-retinal fluid (IRF) at the baseline was associated with poor visual prognosis. Another study showed that 503 out of 989 eyes (50.9%) have discontinued treatment within the 1st year after diagnosis (17). Treatment discontinuation is defined as having a termination visit or lacking a visit during a period of 10–14 months after the first diagnostic visit in the study. A study investigated the recurrence rate within 12 months after the last injection for whom an exit strategy was applied with dry macula on three consecutive visits with an interval of 12 weeks (18). In total, 54 patients (52.9%) showed recurrence during 12 months of follow-up after the last injection in the study. These previous studies reported relatively short-term follow-up results or treatment cessation that was decided by patients.

The purpose of the present study is to investigate the incidence rate of discontinuation of the anti-VEGF injections based on the decision by the treating physicians, the retreatment rate, and the associated risk factors in a large number of patients with nAMD who have been followed up for a long-term period of time.

## Methods

### Design and settings

This study was a single-center retrospective cohort study that is part of the Bundang AMD cohort study (19–21). It was approved by the institutional review board (IRB) of Seoul National University Bundang Hospital (IRB No. B-1910-571-102). This study adhered to the tenets of the Declaration of Helsinki. Informed consent was waived due to the retrospective nature of the study, and the waiver was provided by the IRB of the Seoul National University Bundang Hospital.

### Study participant

We reviewed the medical records of the patients diagnosed with typical nAMD (tnAMD) or polypoidal choroidal vasculopathy (PCV) at the Seoul National University Bundang Hospital from 1 May 2003 to 31 December 2018. Only eyes diagnosed with tnAMD or PCV, treated with one or more anti-VEGF injections, and followed up for 3 years or longer were included in this study. TnAMD was defined based on the fluorescein angiography (FA) and the OCT findings of the macular neovascularization (MNV), as previously described in the ANCHOR and MARINA studies (5, 22). Eyes with PCV were diagnosed according to the criteria described in the EVEREST study report 2 (23): (1) nodular hyperfluorescence of the polypoidal lesion(s) on indocyanine green angiography (ICGA); (2) hypofluorescent halo around the nodule(s); (3) branching vascular network (BVN) supplying the polypoidal lesion(s); and (4) subretinal orange nodules on fundus photography corresponding to the polypoidal lesion(s) on ICGA. The exclusion criteria were as follows: (1) patients who were treated at another hospital prior to their first visit to the Seoul National University Bundang Hospital; (2) patients who returned to our clinic after a follow-up loss for more than a year; (3) patients who refused anti-VEGF injection treatment; and (4) patients with other concomitant pathology that significantly affects central vision such as endophthalmitis, diabetic macular edema, and macular edema associated with retinal vascular diseases.

### Patient evaluation and categorization

**Figure 1:**
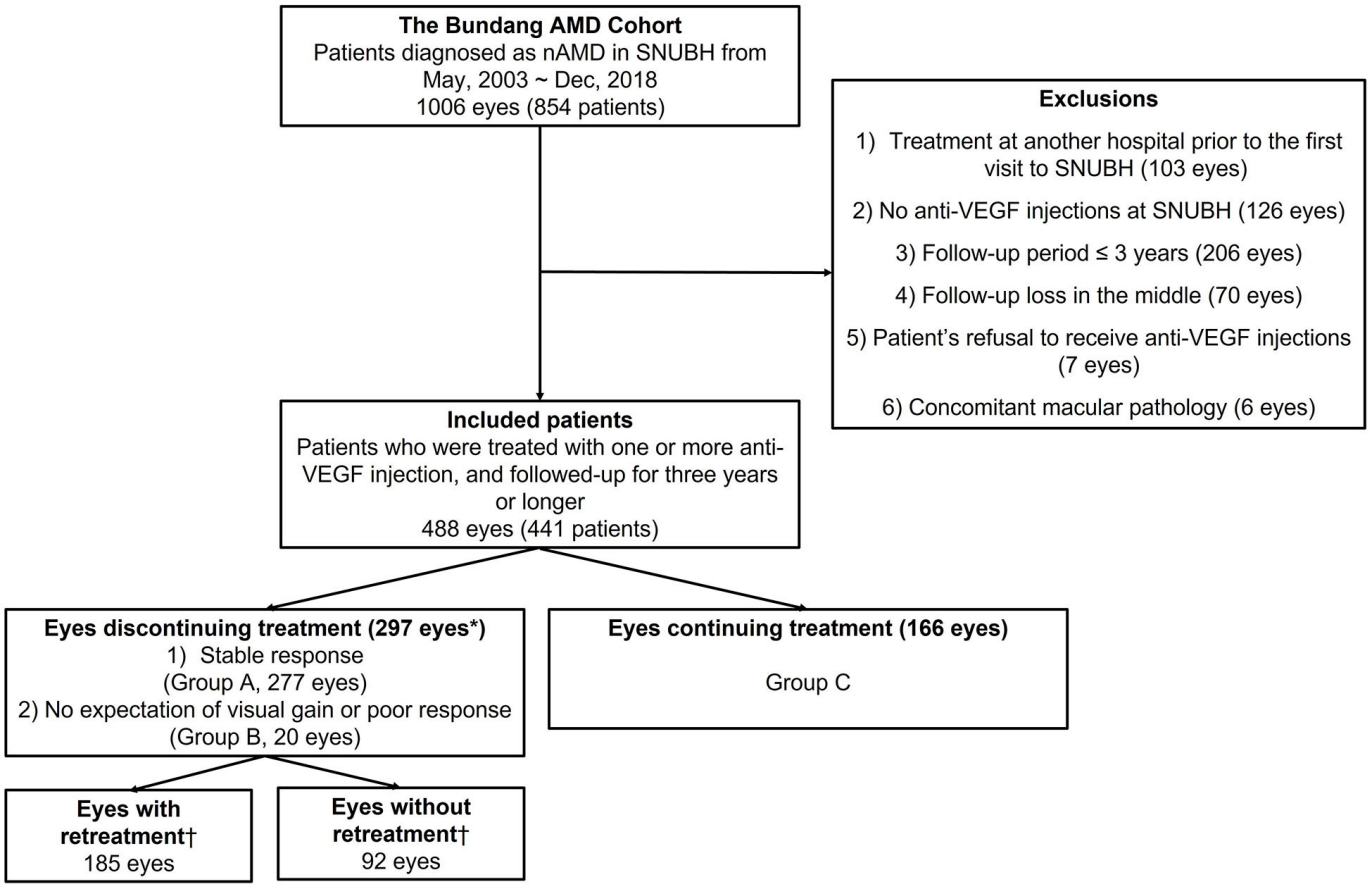
Summary of inclusion flow chart. “Discontinuation of treatment” was defined as the cessation of the anti-VEGF injection for more than 1 year during the follow-up period. *Eyes with vitrectomy within 1 year from the index date (*n* = 9), breakthrough vitreous hemorrhage within 1 year from the index date (*n* = 1), and PDT within 1 year from the index date (*n* = 15) were excluded from “eyes discontinuing treatment.” ^†^Eyes with or without retreatment were defined only for the eyes discontinuing treatment with stable responses (*n* = 277). nAMD, neovascular age-related macular degeneration; SNUBH, Seoul National University Bundang Hospital; VEGF, vascular endothelial growth factor.

A total of 1,006 eyes from 854 patients diagnosed with neovascular AMD from May 2003 to December 2018 were reviewed retrospectively. Of these, 488 eyes from 441 patients were finally included in the analysis after the application of the inclusion and exclusion criteria. A flow chart of the study eyes is presented in Figure 1. The data reviewed from the initial visit include information on the patients' medical history, previous history on ocular management, age, gender, baseline best-corrected visual acuity (BCVA), slit-lamp microscopy, dilated fundus examination, fundus photography (FP), optical coherence tomography (OCT; Spectralis OCT; Heidelberg Engineering Inc, Heidelberg, Germany), FA, and ICGA findings. The clinical course of the eyes included was reviewed from the initial visit to 31 December 2021, including treatments with anti-VEGF injection, photodynamic therapy (PDT), focal laser, pure gas injection for submacular hemorrhage (SMH), and vitrectomy for breakthrough vitreous hemorrhage (VH). “Discontinuation of treatment” was defined as the cessation of the anti-VEGF injection for more than 1 year during the follow-up period. Of the various dates of injection after which treatment is discontinued for more than 1 year, the earliest date of injection is designated as an index date.

With the retrospective review using electronic medical record (EMR), FP, and OCT, the causes of the discontinuation of treatment were categorized into (1) complete resolution; (2) incomplete resolution; (3) no expectation of visual gain; (4) poor response; and (5) intervention other than anti-VEGF injections (vitrectomy or PDT) within 1 year from the index date. Complete resolution was defined as cases without subretinal fluid (SRF), IRF, and exudation from the index date to the date of retreatment for eyes with retreatment (or to the final visit for eyes without retreatment). An incomplete resolution was defined as cases with stable or tolerable mild SRF/IRF from the index date to the date of retreatment (or the final visit). We assumed that the anti-VEGF injection could be discontinued for the cases with “incomplete resolution” in which the treating physician judged that the amount of the fluid is small and stable and that the vision will not be affected. There were no predefined criteria for the tolerable amount of retinal fluid. Study eyes were categorized into eyes with discontinuing treatment and eyes with continuing treatment. Eyes with discontinuing treatment were subdivided into two groups: (1) group A; eyes with stable responses [= complete resolution (group A-1) + incomplete resolution (group A-2)] and (2) group B; those with no expectation of visual gain or poor response. For eyes with continuing treatment (group C), the most recent visit until 31 December 2021 was defined as the last visit. The last visit was regarded as censored data in the survival analysis for the discontinuation of the treatment. Follow-up time was the number of years from the initial injection to the index date or the last visit. For survival analysis of retreatment after the discontinuation of the treatment, only eyes of discontinuing treatment with stable responses were categorized into eyes with or without retreatment. For those without retreatment, the most recent visit was considered the last visit. The last visit was regarded as censored data in the survival analysis of retreatment. The follow-up time was defined as the number of years from the index date to the date of the retreatment or the last visit. The visual acuity (VA) at the first visit, first injection, index date, and last visit were reviewed. The yearly VAs after the first visit were also reviewed. Previously, PDT was mainly performed for subfoveal macular neovascularization (MNV) in eyes with tnAMD or for PCV. Since 2006, the primary treatment for PCV has been changed to intravitreal anti-VEGF injections. Intravitreal anti-VEGF injections were also performed as a primary treatment for tnAMD. Initially, 3 monthly injections of anti-VEGF agents [bevacizumab (1.25 mg/0.05 ml of Avastin, Roche, Basel, Switzerland), ranibizumab (0.5 mg/0.05 ml of Lucentis, Novartis, Basel, Switzerland), or aflibercept (2.0 mg/0.05 ml of Eylea, Bayer, Berlin, Germany)] were given and additional pro re nata (PRN) or treat and extend (TNE) regimens were applied. Brolucizumab (6 mg/0.05 ml of Beovu, Novartis, Basel, Switzerland) injection was also given for some cases with recent follow-up visits.

### Study outcomes

The primary outcomes were the proportion of eyes with discontinuing treatment, the median time to discontinuation of the treatment, the proportion of eyes with retreatment, and the median time to retreatment. The secondary outcome was a visual prognosis among eyes with groups A, B, and C. Additional secondary outcome was the visual prognosis among eyes as follows: (1) eyes discontinuing treatment with complete resolution (group A-1); (2) eyes discontinuing treatment with incomplete resolution (group A-2); and (3) eyes with continuing treatment (group C). Associated risk factors for discontinuing treatment or retreatment were another secondary outcomes. The analysis of risk factors was performed for discontinuing treatment with stable responses (group A) only in order to analyze the factors associated with successful treatment discontinuation in nAMD.

### Statistical analysis

Counts with percentages were used for categorical variables. For continuous variables, mean ± standard deviation (SD) was used. Pearson's chi-square test or Fisher exact test was used to compare the categorical variables between groups. Continuous variables were compared using independent *t*-tests, paired *t*-tests, or one-way analysis of variance (ANOVA). The BCVAs were converted to the logarithm of the minimal angle of resolution (logMAR) value for analysis. The incidence rate of discontinuation of the treatment and retreatment was analyzed using a Kaplan–Meier survival analysis. To investigate the risk factors associated with discontinuing treatment or retreatment, multiple logistic regression analysis was performed for diverse clinical variables including age, gender, unilateral or bilateral involvement, diabetes, hypertension, smoking history, diagnosis (tnAMD or PCV), VA at the initial visit, and the mean number of anti-VEGF injections or PDTs per year for total periods or until index date. In order to assess the visual prognosis among groups, the linear mixed-effect model was applied with the adjustment of age and gender. Statistical analyses were performed using SPSS version 22.0 for Windows (SPSS Inc., Chicago, Illinois, USA), and a *P-*value of < 0.05 was considered statistically significant.

## Results

### Study population, demographic, and clinical characteristics

Demographic and clinical characteristics of the study eyes are presented in Table 1. The discontinuation of treatment was seen in 322 out of 488 eyes (66.0%) during the study period. The presumed causes of the discontinuing treatment were as follows: (1) complete resolution: 203 eyes (63.0%); (2) incomplete resolution: 74 eyes (23.0%); (3) no expectation of visual gain: seven eyes (2.2%); (4) poor response: 13 eyes (4.0%); (5) vitrectomy within 1 year from the index date for SMH, extensive subretinal hemorrhage, breakthrough VH: nine eyes (2.8%); (6) breakthrough VH within 1 year from the index date (with vitrectomy after 1 year from the index date): one eye (0.3%); and (7) PDT within 1 year from the index date: 15 eyes (4.7%). The types and combinations of anti-VEGF drugs used for the study eyes are presented in Supplementary Table 1.

**Table 1:** Demographic and clinical characteristics of the study eyes.

**Characteristics**	**Total eyes**
Number of eyes (*n*)	488 eyes (441 patients)
Age of onset (years), mean ± SD	71.1 ± 8.2 (49–93)
Male: female (*n*, %)	283 (58.0%): 205 (42.0%)
Laterality (unilateral: bilateral; *n*, %)^*^	394 (89.3%): 47 (10.7%)
Diabetes (*n*, %)	216 (44.3%)
Hypertension (*n*, %)	103 (21.1%)
Smoking (smoker [current or ex-smoker]: non-smoker; *n*, %)	251 (51.4%): 237 (48.6%)
Diagnosis (tnAMD: PCV; *n*, %)	334 (68.4%): 154 (31.6%)
Discontinuation of treatment (*n*, %)	322 (66.0%)
Causes for the discontinuation of treatment (*n*, %);	
1) Complete resolution 2) Incomplete resolution 3) No expectation of visual gain 4) Poor response 5) PPV^†^ within 1 year from index date^‡^ 6) Breakthrough VH within 1 year from index date 7) PDT within 1 year from index date	203 [63.0%, (41.6%)] 74 [23.0%, (15.2%)] 7 [2.2%, (1.4%)] 13 [4.0%, (2.7%)] 9 [2.8%, (1.8%)] 1 [0.3%, (0.2%)] 15 [4.7%, (3.1%)]
Follow up period (years), mean ± SD	8.1 ± 3.4 (3.0–18.1)

BCVA, best-corrected visual acuity; PCV, polypoidal choroidal vasculopathy; PDT, photodynamic therapy; PPV, pars planar vitrectomy; SD, standard deviation; TnAMD, typical neovascular age-related macular degeneration; VH, vitreous hemorrhage.

* Unilateral or bilateral involvement was presented for the study patients (n = 441).

† PPV for extensive subretinal hemorrhage, submacular hemorrhage, or breakthrough VH.

‡ Of the various dates of injection after which treatment discontinued for more than 1 year, the earliest date of injection is designated as an index date.

Demographic and clinical characteristics of the eyes with discontinuing treatment and those with continuing treatment are shown in Table 2. The age of onset, gender, diabetes, VA at first injection and at the final visit, VA change, and follow-up period were significantly different among subgroups. The age of onset of group A was significantly younger than that of group B or C (*P* = 0.007). The proportion of female patients was significantly higher in group B than in group A or C (*P* = 0.040). The proportion of diabetes was significantly higher in group B than in group A or C (*P* = 0.047). VA at first injection and at the final visit were significantly worse in group B than in groups A or C (*P* = 0.011, *P* < 0.001, respectively). VA at the final visit was also significantly worse in group A than in group C. VA change was also significantly larger in group B than in group A or C (*P* < 0.001). The follow-up period was significantly shorter in group C than in group A or B (*P* < 0.001). Unilateral or bilateral involvement, hypertension, smoking, diagnosis, and method of treatment were not significantly different among subgroups.

**Table 2:** Demographic and clinical characteristics of the eyes with discontinuing treatment and continuing treatment.

**Characteristics**	**Eyes with discontinuing treatment**		***P*-value (group A vs. B)**	**Eyes with continuing treatment (group C)**	***P*-value (group A vs. C)**	***P*-value (group A vs. B vs. C)**
	**Stable response (group A)**	**No expectation of visual gain or poor response (group B)**				
Number of eyes (*n*, %)	277 (59.8%)	20 (4.3%)	NA	166 (35.9%)	NA	NA
Male: female (*n*, %)	150 (54.2%): 127 (45.8%)	9 (45.0%): 11 (55.0%)	0.428^†^	108 (65.1%): 58 (34.9%)	**0.024^†^**	**0.040** ^†^
Unilateral: bilateral (*n*,%)	223 (80.5%): 54 (19.5%)	13 (65.0%): 7 (35.0%)	0.146^‡^	136 (81.9%): 30 (18.1%)	0.712^†^	0.197^†^
Diabetes (*n*, %)	122 (44.0%)	14 (70.0%)	**0.024^†^**	68 (41.0%)	0.526^†^	**0.047^†^**
Hypertension (*n*, %)	54 (19.5%)	4 (20.0%)	1.000^‡^	43 (25.9%)	0.114^†^	0.281^†^
Smoking (smoker: non-smoker; *n*, %)	133 (48.0%): 144 (52.0%)	9 (45.0%): 11 (55.0%)	0.794^†^	96 (57.8%): 70 (42.2%)	**0.045^†^**	0.114^†^
Diagnosis (tnAMD: PCV; *n*, %)	180 (65.0%): 97 (35.0%)	16 (80.0%): 4 (20.0%)	0.171^†^	121 (72.9%): 45 (27.1%)	0.084^†^	0.117^†^
Method of treatment^§^ (*n*, %)	180 (65.0%): 97 (35.0%)	13 (65.0%): 7 (35.0%)	0.999^†^	104 (62.7%): 62 (37.3%)	0.620^†^	0.882^†^
Retreatment after discontinuing treatment (*n*, %)	185 (66.8%)	6 (30.0%)	**<0.001^†^**	NA	NA	NA
Age of onset (years), mean ± SD	70.3 ± 8.3	72.7 ± 9.9	0.423^*^	72.7 ± 7.6	0.007^*^	**0.007^#^**
VA at first injection (LogMAR), mean ± SD	0.646 ± 0.478	0.907 ± 0.529	0.050^*^	0.574 ± 0.480	0.287^*^	**0.011^#^**
VA at final visit (LogMAR), mean ± SD	0.880 ± 0.764	1.821 ± 0.851	<0.001^*^	0.692 ± 0.541	0.026^*^	**<0.001^#^**
VA change^##^ (LogMAR), mean ± SD	0.234 ± 0.683	0.914 ± 0.803	<0.001^*^	0.118 ± 0.530	0.153^*^	**<0.001^#^**
Follow up period (years), mean ± SD	8.7 ± 3.4	8.9 ± 3.4	0.959^*^	7.0 ± 3.1	<0.001^*^	**<0.001^#^**

NA, non-applicable; LogMAR, logarithm of the minimal angle of resolution; PCV, polypoidal choroidal vasculopathy; SD, standard deviation; TnAMD, typical neovascular age-related macular degeneration; VA, visual acuity. The bold values indicate statistical significance at the *P* < 0.05 level.

* *Post-hoc* analysis of one way analysis of variance (ANOVA).

† Pearson chi-square test.

‡ Fisher exact test.

§ Anti-VEGF only: anti-VEGF + other treatment.

# One way ANOVA.

## VA change (LogMAR) = VA at final visit (LogMAR) – VA at first injection (LogMAR).

Demographics and clinical characteristics of the eyes with and without retreatment after the discontinuation of treatment with stable responses are presented in Table 3. The age of onset, gender, diagnosis, VA at index date, VA change, and follow-up period were significantly different between subgroups. The age of onset was significantly younger in patients with retreatment than in those without retreatment (*P* = 0.008). The proportion of female patients was significantly higher in eyes without retreatment than in eyes with retreatment (*P* = 0.024). The proportion of PCV was significantly higher in the retreatment group (40.5% vs 23.9%, *P* = 0.006). VA at index date was significantly worse in eyes without retreatment than in eyes with retreatment (*P* = 0.029). VA change was significantly larger in eyes with retreatment than in eyes without retreatment (*P* = 0.026). The mean follow-up time from the index date to the retreatment date was 2.97 ± 2.16 years in eyes with retreatment. The mean follow-up time from the index date to the final visit was 6.16 ± 3.28 years in eyes without retreatment. The follow-up period from the initial to the final visit was significantly longer in the retreatment group (*P* = 0.002). Unilateral or bilateral involvement, diabetes, hypertension, smoking, and VA at the final visit were not significantly different between subgroups.

**Table 3:** Demographic and clinical characteristics of eyes with and without retreatment.

**Characteristics**	**Eyes with retreatment**	**Eyes without retreatment**	***P*-value**
Number of eyes (*n*, %)	185 (66.8%)	92 (33.2%)	NA (non-applicable)
Age of onset (years), mean ± SD	69.4 ± 8.2	72.2 ± 8.4	**0.008** ^*^
Male: female (*n*, %)	109 (58.9%): 76 (41.1%)	41 (44.6%): 51 (55.4%)	**0.024** ^†^
Unilateral: bilateral (*n*, %)	170 (81.0%): 40 (19.0%)	88 (78.6%): 24 (21.4%)	0.610^†^
Diabetes (*n*, %)	78 (42.2%)	44 (47.8%)	0.371^†^
Hypertension (*n*, %)	36 (19.5%)	18 (19.6%)	0.983^†^
Smoking (smoker: non-smoker; *n*, %)	95 (51.4%): 90 (48.6%)	38 (41.3%): 54 (58.7%)	0.115^†^
Diagnosis (tnAMD: PCV; *n*, %)	110 (59.5%): 75 (40.5%)	70 (76.1%): 22 (23.9%)	**0.006** ^†^
VA at index date^‡^ (LogMAR), mean ± SD	0.596 ± 0.509	0.762 ± 0.627	**0.029** ^*^
VA at final visit (LogMAR), mean ± SD	0.876 ± 0.748	0.888 ± 0.799	0.904^*^
VA change^§^ (LogMAR), mean ± SD	0.280 ± 0.605	0.126 ± 0.504	**0.026** ^*^
Time from index date to retreatment (or final visit; years), mean ± SD	2.97 ± 2.16 (index date–retreatment date)	6.16 ± 3.28 (index date–final visit)	**< 0.001** ^*^
Follow up period (years), mean ± SD	9.1 ± 3.5	7.8 ± 3.2	**0.002** ^*^

LogMAR, logarithm of the minimal angle of resolution; PCV, polypoidal choroidal vasculopathy; TnAMD, typical neovascular age-related macular degeneration; SD, standard deviation; VA, visual acuity. The bold values indicate statistical significance at the *P* < 0.05 level.

* Independent t-test.

† Pearson chi-square test.

‡ Of the various dates of injection after which treatment discontinued for more than 1 year, the earliest date of injection is designated as an index date.

§ VA change (LogMAR) = VA at final visit (LogMAR) – VA at index date (LogMAR) .

### Discontinuation of treatment and retreatment

**Figure 2:**
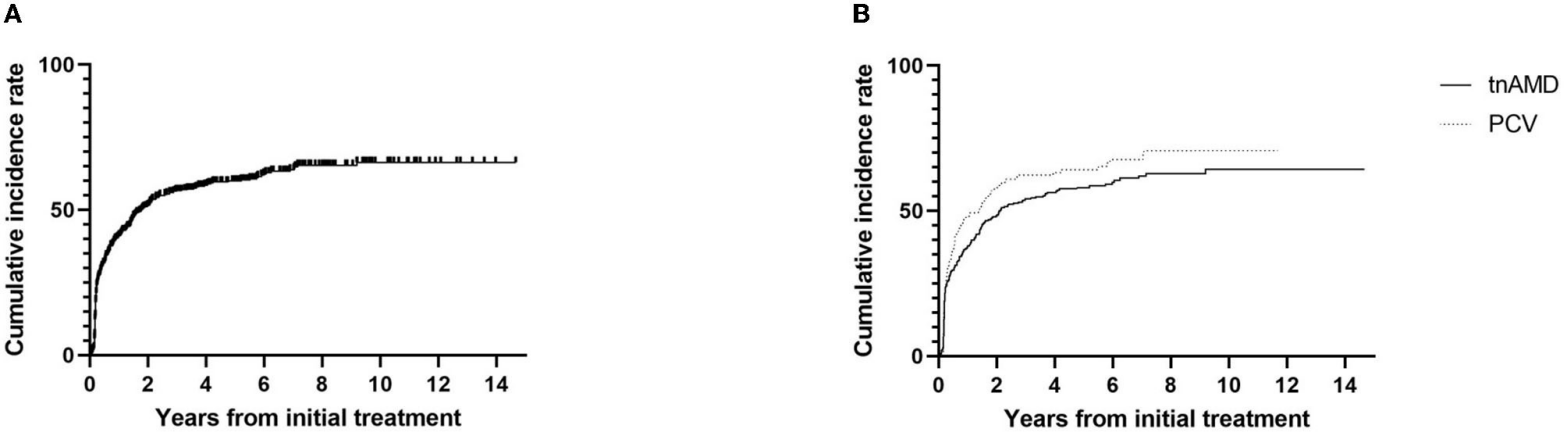
Kaplan–Meier survival curve of the cumulative incidence for the discontinuation of the treatment. **(A)** The cumulative incidence for the discontinuation of the treatment for total eyes (*n* = 463). **(B)** The cumulative incidence for the discontinuation of the treatment according to subtypes of neovascular age-related macular degeneration [typical neovascular age-related macular degeneration (*n* = 317) vs. polypoidal choroidal vasculopathy (*n* = 146)]. TnAMD, typical neovascular age-related macular degeneration; PCV, polypoidal choroidal vasculopathy.

Among the 297 eyes with discontinuing treatment, 277 eyes (59.8%) were those with stable responses (group A). The other 20 eyes (4.3%) were those with no expectation of visual gain or poor response (group B; Table 2). The discontinuation of the treatment with stable responses (group A) was 56.8% (277/488) of the total eyes. Kaplan–Meier curve showing the time to the discontinuation of the anti-VEGF injections is presented in Figure 2A. The median time to the discontinuation of treatment was 1.5 years after the initial injection. Survival analysis in total eyes (*n* = 463) revealed that the incidence rates of discontinuation of the treatment were 44.1, 54.1, 62.0, and 68.1% of eyes within 1, 2, 5, and 10 years after the initial injection, respectively. Kaplan–Meier curve showing the time to the discontinuation of the anti-VEGF injections in eyes with tnAMD and PCV is also shown in Figure 2B. The incidence rates of the discontinuation of treatment were not significantly different between eyes with tnAMD and PCV (log-rank test, *P* = 0.117). Among the eyes with discontinuing treatment, the proportion of eyes with stable responses (group A) was not significantly different between PCV (97/101 = 96.0%) and tnAMD (180/196 = 91.8%, *P* = 0.171; Table 2). The incidence rates of the cessation of treatment with stable responses were not significantly different between eyes with tnAMD and PCV (*P* = 0.222). The incidence rates of the suspension of the treatment with no expectation of visual gain or poor response were not significantly different between eyes with tnAMD and PCV (*P* = 0.178). Among patients with bilateral involvement, there was no significant difference in the time from initial injection to the discontinuation of treatment between the first treated and the second eyes (*P* = 0.846, paired *t*-test).

**Figure 3:**
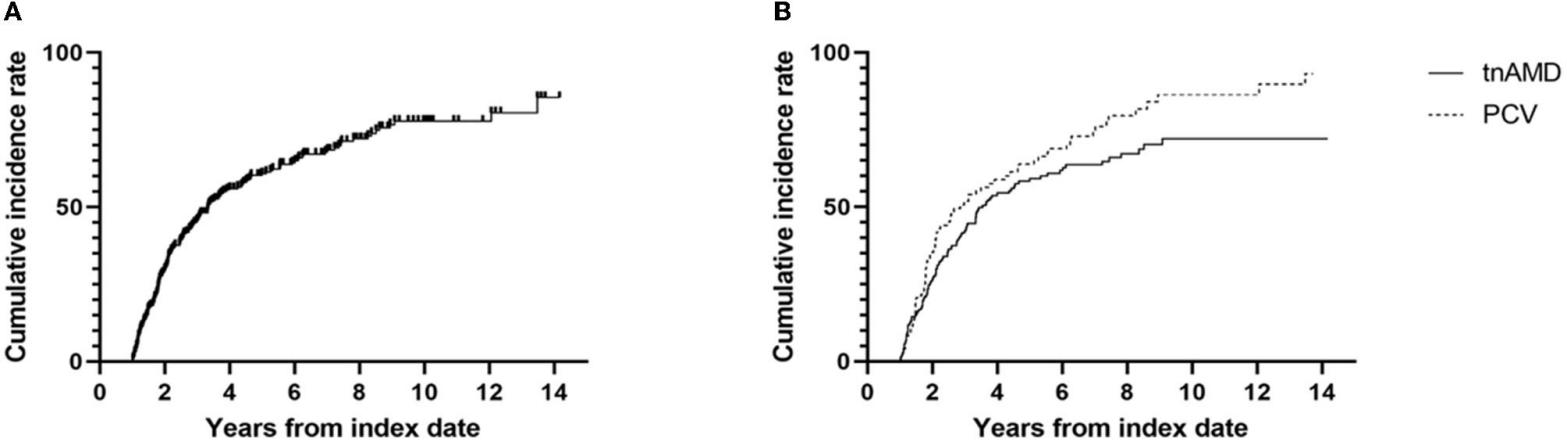
Kaplan–Meier survival curve of the cumulative incidence for the retreatment. **(A)** The cumulative incidence for the retreatment of eyes discontinuing treatment with stable responses (*n* = 277). **(B)** The cumulative incidence for the retreatment of eyes discontinuing treatment with stable responses according to the subtypes of neovascular age-related macular degeneration [typical neovascular age-related macular degeneration (*n* = 180) vs. polypoidal choroidal vasculopathy (*n* = 97)]. TnAMD, typical neovascular age-related macular degeneration; PCV, polypoidal choroidal vasculopathy.

The retreatment after the discontinuation of treatment with stable responses was noted in 185 out of 277 eyes (66.8%; Table 3). Kaplan–Meier curve showing time to retreatment after the cessation of the injections is presented in Figure 3A. The median time to the retreatment was 3.3 years after the index date of treatment discontinuation. Survival analysis in eyes of discontinuing treatment with stable responses (*n* = 227) revealed that the incidence rates of the retreatment were 29.6, 60.8, and 77.8% of eyes within 2, 5, and 10 years after the index date, respectively. Kaplan–Meier curve showing retreatment in eyes with tnAMD and PCV is also shown in Figure 3B. The incidence rates of the retreatment were significantly higher in PCV than in tnAMD (log-rank test, *P* = 0.043). The median time to the retreatment since the index date was 2.9 years in PCV and 3.5 years in tnAMD. Among patients with bilateral involvement, there was no significant difference in the time from the discontinuation of treatment to retreatment between the first treated and the second eyes (*P* = 0.065, paired *t*-test).

### Visual outcomes

**Figure 4:**
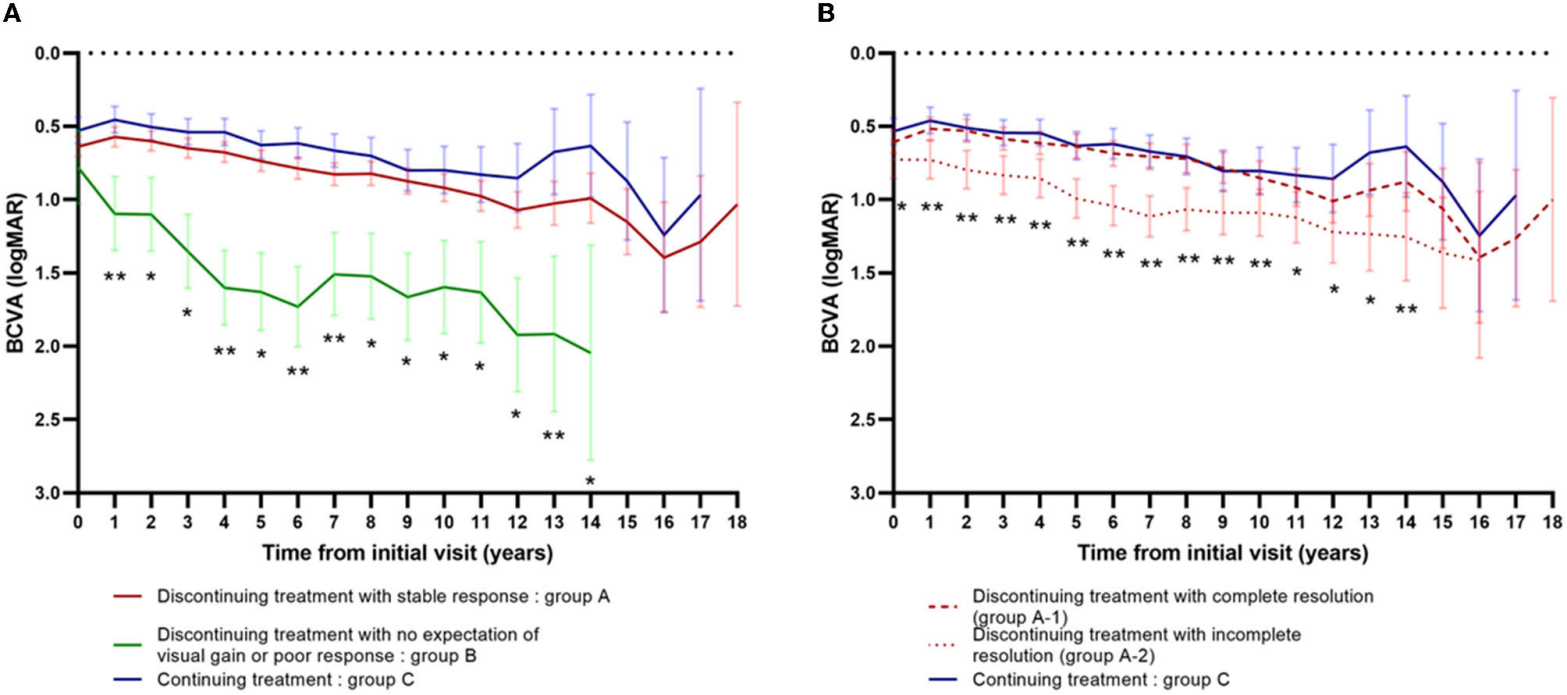
Changes in visual acuity from the initial visit among subgroups. **(A)** Changes in visual acuity among group A (*n* = 277) vs. group B (*n* = 20) vs. group C (*n* = 166). *Significantly different between group B vs. group A and group B vs. group C. **Significantly different between group B vs. group A, group B vs. group C, and group A vs. group C. **(B)** Changes in visual acuity among group A-1 (*n* = 203) vs. group A-2 (*n* = 74) vs. group C (*n* = 166). *Significantly different between group A-2 vs. group C. **Significantly different between group A-2 vs. group C and group A-2 vs. group A-1.

Changes in visual acuity from the initial visit among subgroups are presented in Figure 4. Changes in visual acuity among groups A, B, and C are presented in Figure 4A. Using the linear mixed model with the adjustment of age and gender, the BCVA was found to be significantly worse in group B compared to group A or C, at each time point from 1 to 14 years after the initial visit (*P* < 0.001, annually from 1 to 12 years; *P* = 0.002 between group B and A, *P* < 0.001 between group B and C at 13 years, respectively; *P* = 0.006 between group B and A, and *P* = 0.001 between group B and C at 14 years, respectively). At 1, 4, 6, 7, and 13 years after the initial visit, the BCVA was also significantly worse in group A than group C (*P* = 0.040, *P* = 0.018, *P* = 0.008, *P* = 0.020, and *P* = 0.035, respectively).

Changes in visual acuity among groups A-1, A-2, and C are presented in Figure 4B. Using the linear mixed model with the adjustment of age and gender, the BCVA was significantly worse in group A-2 than group C, at each time point from the baseline to 14 years after the initial visit (*P* < 0.001, annually from 2 to 8 years; *P* = 0.016 at baseline, *P* = 0.001 at 1 year, *P* = 0.007 at 9 years, *P* = 0.013 at 10 years, *P* = 0.026 at 11 years, *P* = 0.023 at 12 years, *P* =0.004 at 13 years, and *P* = 0.008 at 14 years, respectively). At 1 to 10 years and at 14 years after the initial visit, the BCVA was also significantly worse in group A-2 than group A-1 (*P* = 0.007 at 1year, *P* = 0.001 at 2 and 3 years, *P* = 0.002 at 4 years, *P* < 0.001 at 5 to 8 years, *P* = 0.001 at 9 years, *P* = 0.018 at 10 years, and *P* = 0.040 at 14 years, respectively).

The mean BCVA of eyes with retreatment was significantly better than those without retreatment at the index date (*P* = 0.029), but it was similar to eyes without retreatment at the final visit (*P* = 0.904; Table 3).

### Risk of discontinuation of treatment and retreatment

The results of multiple logistic regression analysis performed to determine the clinical factors associated with the discontinuation of the treatment and retreatment are shown in Table 4. PCV and lower annual mean number of injections were factors significantly associated with the discontinuation of the treatment [OR = 2.547 (95% CI; 1.304–4.974), *P* = 0.006; OR = 0.264 (95% CI; 0.209–0.335), *P* < 0.001, respectively]. Younger age, male gender, and PCV were significantly associated factors for retreatment [OR = 0.966 (95% CI; 0.936–0.998), *P* = 0.038; OR = 1.809 (95% CI; 1.079–3.034), *P* = 0.025; OR = 1.880 (95% CI; 1.048–3.371), *P* = 0.034, respectively]. PDT was significantly more frequently performed in PCV than in tnAMD [49.4% (=76/154) vs. 22.8% (=76/334), *P* < 0.001].

**Table 4:** Clinical factors associated with discontinuation of the treatment or retreatment.

**Clinical factors**	**Odds ratio (OR)**	**95% CI of OR**	***P*-value**
**A. Risk of the discontinuation of the treatment** ^*^			
PCV (vs. tnAMD)	2.547	(1.304–4.974)	**0.006**
Annual mean number of injections	0.264	(0.209–0.335)	**< 0.001**
**B. Risk of retreatment** ^†^			
Age	0.966	(0.936–0.998)	**0.038**
Gender (male)	1.809	(1.079–3.034)	**0.025**
PCV (vs. tnAMD)	1.880	(1.048–3.371)	**0.034**

CI, confidence interval; PCV, polypoidal choroidal vasculopathy; PDT, photodynamic therapy; TnAMD, typical neovascular age-related macular degeneration; VA, visual acuity. The bold values indicate statistical significance at the *P* < 0.05 level.

* Multiple logistic regression analysis was applied. Predictive factors were simultaneously entered into a backwards stepwise model selection process: age at diagnosis, gender, unilateral or bilateral involvement, diabetes mellitus, hyper tension, smoking history (including previous and current smokers), PCV, VA at first visit, mean number of PDTs/year for the total periods, and mean number of injections/year for the total periods.

† Multiple logistic regression analysis was applied. Predictive factors were simultaneously entered into a backwards stepwise model selection process: age at diagnosis, gender, unilateral or bilateral involvement, diabetes mellitus, hyper tension, smoking history (including previous and current smokers), PCV, VA at first visit, mean number of PDTs/year until index date, and mean number of injections/year until index date.

## Discussion

We investigated the real-world incidence rate of the discontinuation of the anti-VEGF, the subsequent retreatment rate, and the associated risk factors in a large number of patients with nAMD who were followed up for 8 years on average. In the present study, the discontinuation of treatment in nAMD patients, which does not exist in the randomized clinical trial (RCT), was considered and analyzed in a real-world setting. Our study found that 66% of eyes with nAMD discontinued the anti-VEGF injections. Of the eyes with discontinuing treatment with a stable response, 67% required retreatment. The median time to the treatment suspension was 1.5 years after the initial injection. The significantly associated factors for the discontinuation were PCV and the lower annual number of injections. The median time to retreatment was 3.3 years after the cessation of the treatment. The associated factors for the retreatment were younger age, male gender, and PCV.

There was no significant difference in the time to the discontinuation of treatment or retreatment between the first treated eyes and the fellow eyes among patients with bilateral involvement. In addition, there was no difference between the eyes with unilateral involvement and those with bilateral involvement. Therefore, the results were analyzed based on the eyes. In our study, 322 of 488 eyes (66.0%) with nAMD experienced the discontinuation of the anti-VEGF injections during the study period. The median time to discontinuation was 1.5 years after the initial injection. One previous study reported that 207 of 272 patients (76.1%) experienced the discontinuation of the anti-VEGF therapy (24). Ceasing the intravitreal injections for ≥6 months in the study was defined as the discontinuation of the anti-VEGF. The study showed that 185 of 272 patients (68.0%) discontinued the treatment due to the doctor's decision and the remaining 22 patients (8.1%) suspended the injections due to their own decision (“no deterioration in vision,” “financial burden,” and “ineffective treatment”). Our study found a slightly lower rate of discontinuation of the anti-VEGF treatment, which was defined as the suspension of the injections for more than a year. In this study, the patient's refusal of the injection was excluded from the cases (Figure 1). Our previous report revealed that the mean number of injections per year for nAMD was ≤2 from the 2nd year of injections in contrast to the RCT (21). We infer that the relatively small number of injections of ≤2 was related to the discontinuation of the anti-VEGF injections.

We found that 185 out of 277 eyes (66.8%) with discontinuing treatment had retreatment after the discontinuation with a stable response. The median time to retreatment was 3.3 years after the cessation of the treatment. Aslanis et al. (18) reported the recurrence rate within 12 months after the last injection with a TNE regimen. They prospectively included the patients with dry macula on three consecutive visits/injections 12 weeks apart with a TNE regimen. The eligible patients received another aflibercept injection at the baseline and were followed up at 4 months and then bimonthly, with the last visit 12 months after the last injection. A total of 53% (=54/102) of patients showed recurrence after 12 months of follow-up in the study. The mean time to recurrence was 6.7 ± 2.2 months in the study. Another recent study reported that the recurrence rate was 33% at 1 year after treatment cessation (25). The result of the current study is slightly different from those of previous studies in that our study analyzed the rate of retreatment instead of recurrence. The discontinuation of the treatment was also analyzed based on a pre-specified definition in this study. Our longer median time to retreatment may be because our study included relatively more patients with favorable courses compared to those previous reports. Considering that the median time to retreatment after the discontinuing treatment was about 3 years in this study, long-term regular follow-up is needed after the discontinuation of treatment in nAMD patients. Out of 277 eyes discontinuing treatment with a stable response, 92 eyes (33.2%) did not have retreatment during the study period. This represents 18.9% of the total study eyes (92/488). We infer that most of those 92 eyes were not given retreatment due to no recurrence with a stable response.

In this study, the BCVA was not significantly different between eyes discontinuing treatment with complete resolution (group A-1) and those continuing treatment (group C) in the overall period. The result implies that the visual prognosis is similar between eyes discontinuing treatment with complete resolution and those continuing treatment. The proportion of retreatment after the cessation of the treatment was 64% in group A-1 and 30% in group B. The relatively higher incidence of retreatment of 64% in group A-1 may influence the overall visual prognosis similar between groups A-1 and group C.

Furthermore, the BCVA was significantly worse in group A-2, compared to group C and A-1, in most of the follow-up period. The result showed that the visual prognosis of the eyes with no fluid was significantly better than the eyes with mild tolerable fluid in the cases of treatment suspension for longer than 1 year. This also implies that the complete resolution of retinal fluid is beneficial for better visual outcomes in the long term. Some physicians target no fluid, while others tolerate mild residual fluids in the treatment of nAMD. The FLUID study revealed that the mild (≤200 um) SRF was not detrimental to the visual outcomes for up to 2 years (26). The Comparison of Age-related Macular Degeneration Treatments Trials (CATTs) *post-hoc* analysis showed that residual SRF was associated with better visual outcomes compared to no SRF during 5 years of treatment (27). However, our real-world study resulting from about 8 years of follow-up suggests that eyes with a mild stable fluid have an inferior visual outcome compared to eyes without fluid at all time points. Further research is needed to reach a decisive conclusion on this issue.

In our study, the BCVA was significantly poor in group B, compared to group A or C, at each time point from 1 to 14 years after the initial visit. The BCVA at the first injection was significantly worse in group B, compared to group A or C (Table 2). We infer that the initial BCVA may affect the final visual prognosis in nAMD patients. One previous report showed that poor baseline VA was associated with poor VA at 5 years after treatment with bevacizumab or ranibizumab in the cohort of patients enrolled in Comparison of Age-related Macular Degeneration Treatments Trials (CATTs) (28). Westborg et al. also showed that better baseline VA was associated with reduced risk of VA loss in nAMD patients (29). Group B included patients with eyes of discontinuing treatment with no expectation of visual gain or poor response. Therefore, the visual prognosis of group B is expected to be poor. The proportion of retreatment after cessation of treatment was also significantly lower in group B (6/20, 30%) than in group A (185/277, 67%; Table 2).

In this study, among eyes discontinuing treatment with a stable response, the mean BCVA of eyes with retreatment was better than those without retreatment at the index date but similar to eyes without retreatment at the final visit (Table 3). The result implies that the long-term final VA becomes similarly poor regardless of the retreatment for the eyes with discontinuing injections for nAMD patients. Kim et al. revealed that marked deterioration in VA was found in nAMD patients discontinuing treatment (16). The mean logMAR of BCVA at treatment discontinuation and at 24 months was 1.02 ± 0.20 and 1.60 ± 0.56, respectively (*P* < 0.001) in their study. Similarly, the BCVA deteriorated at the final visit compared to the index date in eyes with discontinuing treatment regardless of the retreatment in this study.

PCV and the lower mean number of injections per year were significantly associated clinical factors with the discontinuation of the anti-VEGF injections in this study. The natural course of PCV is variable. It may be relatively stable, or there may be repeated bleeding and leakage with vision loss and chorioretinal atrophy (30). In our study, a higher rate of treatment suspension in PCV might imply a relatively stable disease course in PCV compared to tnAMD. PCV showed a longer interval and lower rate of recurrence after PDT (31). The increased risk of the discontinuation of anti-VEGF in PCV compared to tnAMD in this study may be associated with the fact that combination therapy with PDT was applied for nearly half of the eyes (76/154; 49.4%) with PCV. We also found that as the mean number of injections per year increases, the odds ratio of the discontinuation of the treatment decreases. The total and the mean number of injections become larger in patients with continuing treatment than in those with discontinuing treatment. Therefore, as the average number of injections per year increases, the discontinuation of the treatment becomes less likely. It is not a causal relationship but rather an association.

In this study, younger age, male gender, and PCV were significantly associated with the risk of retreatment. Geographic atrophy (GA) is recognized clinically as sharply circumscribed areas of the retinal pigment epithelium (RPE) loss through which the underlying choroidal vessels become visible (30). Age and genetic components are the significant risk factors for GA (32). As the age increases, the incidence rate of the GA becomes higher and the visual function gets more deteriorated due to the loss of the RPE and the photoreceptor. It becomes harder to expect visual improvement with the retreatment in older patients. Therefore, older age can be an associated factor for the less risk of retreatment.

There have been few studies comparing the rate of recurrence (or retreatment) according to gender in nAMD. One study investigated the predictive factors associated with recurrence after 3 monthly loading doses of intravitreal ranibizumab (33). Patients were followed up for more than 1 year after the first injection by an as-needed regimen. Older patients (*P* = 0.006) and male patients (*P* = 0.018) tended to require re-treatment for recurrence during the 1st year, yet the statistical significance disappeared when evaluated for 2 years in the study. Further research is needed to reveal the association between male gender and risk of retreatment.

PCV was significantly associated with the risk of retreatment in this study. This result is associated with a relatively higher incidence of recurrence of PCV compared to tnAMD. There have been a few studies comparing the long-term recurrence rate between tnAMD and PCV. One study reported that the incidence of lesion reactivation was higher in patients with PCV than in those with tnAMD, especially after 12 months (34). In another study, of the 10 baseline factors evaluated, the subtype of AMD was the sole significant factor associated with the interval to the recurrence after the 3 monthly loading injections of the ranibizumab (33). The interval to the recurrence was significantly shorter in eyes with PCV (3.6 ± 2.2 months) than in eyes with tnAMD (4.5 ± 2.2 months, *P* = 0.015).

This study has several limitations. First, this study was a retrospective design and had an inherent risk of selection bias. Second, the treatment schedules were not standardized and differed among patients. The PRN treatment regimen was applied for most of the patients before December 2017. The Korean national health insurance covered only 14 injections of ranibizumab and/or aflibercept in total for nAMD patients before December 2017. This limit on the number of injections covered by insurance was lifted afterward. In recent years, the TNE regimen has been the mainstay treatment strategy. In addition, the treatment modality was not randomly applied but was chosen at the discretion of physicians. PDT had been applied for subfoveal CNV in tnAMD or PCV unresponsive to anti-VEGF treatment. Long-term prospective studies with well-controlled and standardized treatment regimens are needed to investigate the incidence and risk factors of the discontinuation of anti-VEGF injections and retreatment. Nevertheless, the strength of this study is that it investigated the long-term cumulative incidence of the discontinuation of the treatment and retreatment for real-world nAMD patients, and it included a large number of patients who were followed up for more than 8 years on average.

## Conclusion

Two-thirds of eyes with nAMD had the discontinuation of the anti-VEGF injections and two-thirds of eyes discontinuing treatment with stable responses experienced retreatment in the real world. PCV was a significantly associated factor for the discontinuation of the treatment with stable responses and retreatment. Therefore, long-term follow-up and regular close monitoring are needed to evaluate the recurrence and the need for retreatment in nAMD patients.

## Data availability statement

The datasets used and/or analysed during the current study are available from the corresponding author on reasonable request.

## Ethics statement

This retrospective study was approved by the institutional review board (IRB) of Seoul National University Bundang Hospital (IRB No. B-1910-571-102). This study adhered to the tenets of the Declaration of Helsinki.

## Author contributions

SC and SW: study design, data analysis, and interpretation. SC: writing the first draft of the manuscript. SC, KP, SP, KJ, and SW: data collection and manuscript review. All authors read and approved the final version of the manuscript.
