# Supplementary material for: Discontinuation of treatment and retreatment of neovascular age-related macular degeneration in the real-world: Bundang AMD cohort study report 5
Source: Front Med (Lausanne). 2023 Jul 10;10:1204026. doi: 10.3389/fmed.2023.1204026 (PMC10364640; doi:10.3389/fmed.2023.1204026)
Supplement: Supplementary file 1 [file Table_1.docx]

**Supplementary table 1. Types and combinations of anti-VEGF drugs used for study eyes**

| **Types of anti-VEGFs** | **Number of cases (n)** | **Proportion (%)** |
| --- | --- | --- |
| Bevacizumab | 52 | 10.7% |
| Ranibizumab | 67 | 13.7% |
| Aflibercept | 30 | 6.1% |
| Bevacizumab + Ranibizumab | 138 | 28.3% |
| Bevacizumab + Aflibercept | 47 | 9.6% |
| Ranibizumab+ Aflibercept | 19 | 3.9% |
| Aflibercept + Brolucizumab | 1 | 0.2% |
| Bevacizumab + Ranibizumab + Aflibercept | 116 | 23.8% |
| Ranibizumab + Aflibercept + Brolucizumab | 2 | 0.4% |
| Bevacizumab + Aflibercept + Brolucizumab | 3 | 0.6% |
| Bevacizumab + Ranibizumab + Aflibercept + Brolucizumab | 13 | 2.7% |
| Total | 488 | 100% |
